# Risk of Axillary Nerve Injury With Medial Support Screws in Intramedullary Nails: An Anatomical Study

**DOI:** 10.7759/cureus.64119

**Published:** 2024-07-08

**Authors:** Shunsuke Kobayashi, Satoshi Miyake, Terufumi Shibata, Kei Matsunaga, Naofumi Hata, Teruaki Izaki, Takuaki Yamamoto

**Affiliations:** 1 Department of Orthopaedic Surgery, Faculty of Medicine, Fukuoka University, Fukuoka, JPN; 2 Department of Orthopaedic Surgery, Fukuoka University Chikushi Hospital, Chikushino, JPN

**Keywords:** axillary nerve branch, proximal humeral fracture, medial support screw, cadaveric study, axillary nerve injury, intramedullary nail

## Abstract

Background: In recent years, intramedullary nails with medial support screws for proximal humeral fractures have become available. Although these devices have a potential risk of iatrogenic axillary nerve injury, no studies have investigated the anatomical relationship between the medial support screws in the modern intramedullary nail and the axillary nerve. This study aimed to clarify the anatomical relationship between the medial support screws in the intramedullary nail and the axillary nerve.

Materials and methods: In total, 29 cadaveric shoulders (mean age: 82.6 years old (range: 61-105); 15 males and 14 females) were included in this study. Shoulders within whole-body cadavers were used in all cases. A single proximal humeral nail with medial support screws (ARISTO Proximal Humeral Nail; MDM, Tokyo, Japan) was used. The distance of each medial support screw from the axillary nerve and its branches was measured.

Results: In two (6.90%) of 29 shoulders, the axillary nerves came into contact with the medial support screws. In the remaining 27 of 29 shoulders (93.1%), the nerves were located proximal to the medial support screws.

Conclusion: Medial support screws in proximal humeral fracture nails had the potential to injure the axillary nerve and its branches.

## Introduction

The modern locking plate with medial support screws has had a significant positive impact on the clinical outcomes of proximal humerus fractures [1-5]. However, the medial support screws in the plate have a potential risk of iatrogenic axillary nerve injury [6-9]. Several anatomical studies have tried to address this risk by clarifying the anatomical relationship between the medial support screws in the plate and the axillary nerve [10-12].

The proximal humerus fracture nail with medial support screws has recently been developed. The modern intramedullary nail and the plate have had a significant positive impact on the surgical treatment of proximal humeral fractures. On the other hand, the device may also have a potential risk of iatrogenic axillary nerve injury. However, no anatomical studies have investigated the anatomical relationship between the medial support screw in the modern intramedullary nail and the axillary nerve.

This study aimed to clarify the anatomical relationship between the medial support screws in the intramedullary nail and the axillary nerve. We hypothesized that the medial support screws in the intramedullary nail would have a potential risk of iatrogenic axillary nerve injury. This study was conducted following the Declaration of Helsinki and was approved by our institutional review board (approval number U23-04-013).

## Materials and methods

All the cadavers used in this study were donated to our institution. The donors voluntarily indicated their willingness to donate their bodies for anatomical education and study. This system is established in Japan’s Law on Body Donation for Medical and Dental Education. Our study is fully compliant with this law.

All cadavers (n=29 shoulders) used for dissection practice for students of the Faculty of Medicine of Fukuoka University in 2023 were included in this study. Specimens with marked deformities of the shoulder joint and surgically treated cadavers were excluded (n=0). Finally, a total of 29 shoulders (mean age: 82.6 years (range: 61-105); 15 men and 14 women; 25 left shoulders and one right shoulder) of 29 adult Japanese cadavers were measured in this study. All measurements in this study were made using shoulders within whole-body cadavers. To avoid students damaging the shoulders of corpses used for research purposes, our institution has established a rule that the right shoulders are for students and the left shoulders are for research. As a result, mostly left shoulders were included in this research. Cadavers were fixed in 8% formaldehyde and preserved in 30% ethanol. The dissections were performed by orthopedic surgeons 10 years postgraduation, assisted by two orthopedic specialists.

First, the cadaver was placed in the supine position, and humeral length (distance between the lateral end of the acromion and the lateral epicondyle of the humerus) was measured from the body surface [13]. The anterior scapulohumeral joint was developed using a deltopectoral approach. The skin incision was extended to the clavicle, and the clavicle was osteotomized while preserving the deltoid attachment. The trapezius was detached from the supraclavicular attachment. Next, a pillow block was placed under the scapula, and the cadaver was placed in a semi-supine position to facilitate observation of the posterior aspect of the shoulder.

The trapezius muscle attached from the acromion and the scapular spine was detached from the origin. The posterior deltoid muscle attached to the scapular spine was identified, and the scapula was osteotomized at the scapular spine. By detaching the coracoclavicular ligament from the inferior aspect of the clavicle and the coracoacromial ligament from the inferior aspect of the acromion, the deltoid muscle was freed in a single mass with the osteotomized clavicle and scapular spine. The deltoid muscle was carefully flipped, and the anterior axillary nerve could be identified with its adhesion to the fascia of the deltoid muscle. The reasons for osteotomizing the clavicle and scapular spine with the deltoid muscle attachment preserved are as follows. The reduction of the osteotomy site would restore the flipped deltoid muscle to its original position, thereby accurately recreating the native position of the anterior axillary nerve behind the deltoid muscle. The conjoint tendon was detached from the coracoid process attachment, and the anterior capsule was detached with the subscapularis tendon to keep the glenohumeral joint in an intermediate position. The supraspinatus, infraspinatus, and teres minor tendons were detached from the humeral attachments to expose the humeral head.

**Figure 1:**
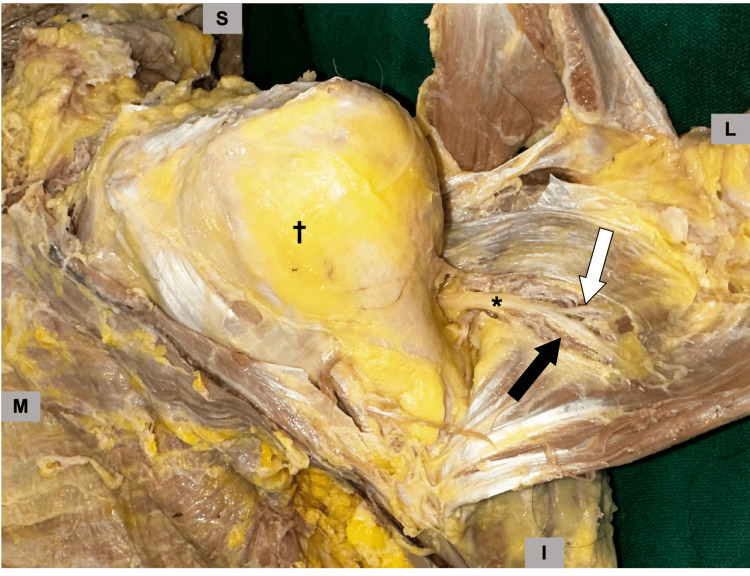
Anatomical location of the anterior axillary nerve and its branches Left shoulder viewed from the anterior with the clavicle and scapular spine rotated while preserving the deltoid attachments and exposing the humeral head. At the right side of the humeral head is the rotated deltoid muscle. †: humeral head,: pre-branched anterior axillary nerve (pre-AAN), black arrow: post-branched anterior axillary nerve (post-AAN), white arrow: ascending axillary nerve branch (ascending-AAN)

The course of the axillary nerve and its branches were recorded. The distribution of the anterior axillary nerve was observed in detail. An ascending branch was observed at the end of the anterior branch of the axillary nerve. This was defined as an ascending axillary nerve branch (ascending-AAN). The proximal part of the branch was defined as the pre-branched anterior axillary nerve (pre-AAN) and the distal part as the post-branched anterior axillary nerve (post-AAN) (Figure 1). Each branch was marked with a pin.

**Figure 2:**
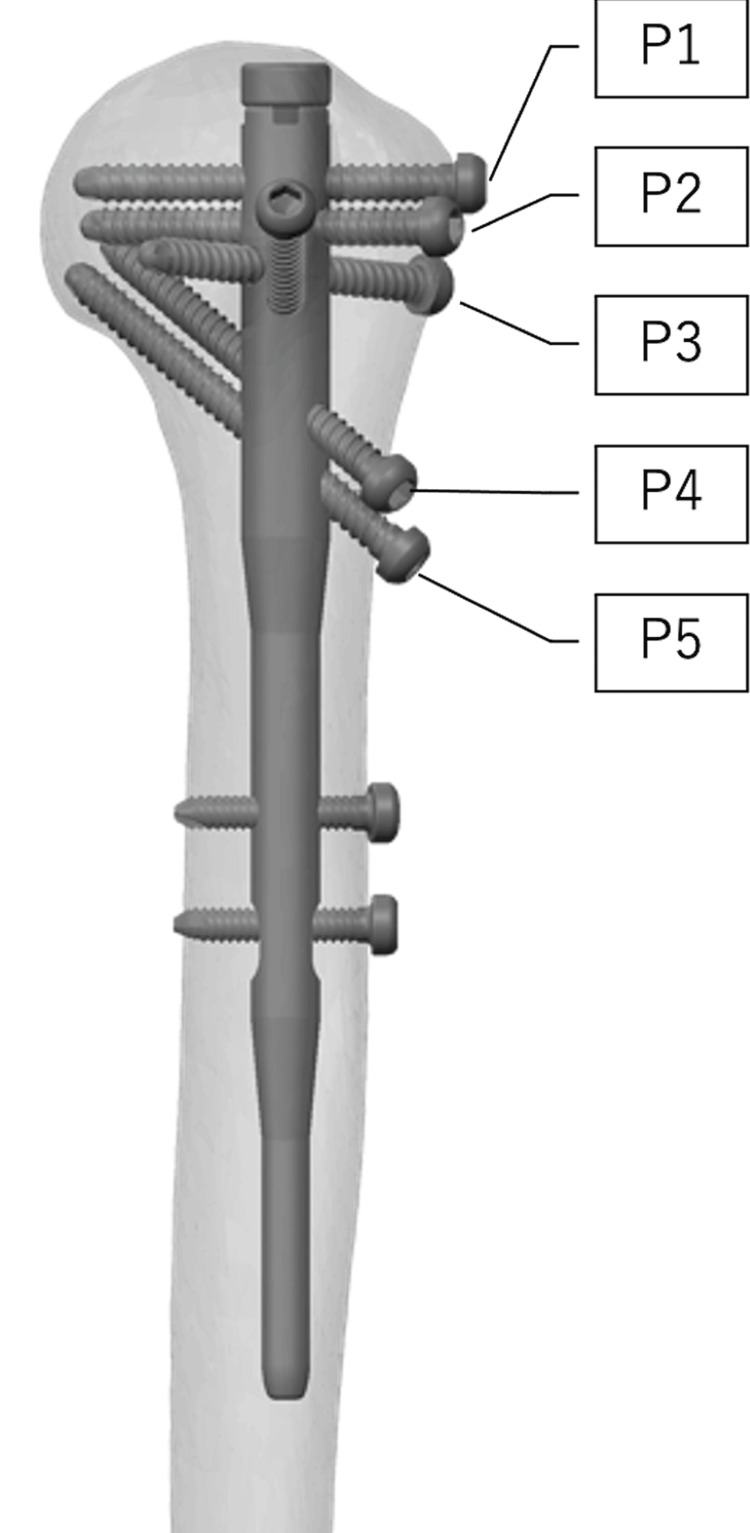
Position of the screw insertion in the ARISTO Nail P1-P3 are proximal locking screws, while P4 and P5 are locking screws that support the inferomedial humeral cortical bone. This figure is permitted by MDM, Japan.

In this study, ARISTO Proximal Humeral Nails (MDM, Tokyo, Japan) were used as nails with medial support screws. This device is designed for optimal screw insertion in the smaller East Asian skeleton based on 3D-CT analysis of humeral head morphology. Up to six proximal locking screws can be inserted (P1, P2, P3: LM directional screws, P4, P5: medial support screw, AP: AP directional screws). The total length of the intramedullary nails was 150 mm. The distance from the upper edge of the intramedullary nail to each screw hole was P1: 9 mm; P2: 14 mm; P3: 19 mm; P4: 33.5 mm; and P5: 40.5 mm (Figure 2). The intramedullary nail was inserted while preserving the limb’s position as in the actual surgical procedure. The nail was inserted until the top of the implant was aligned with the osteochondral cartilage under direct visualization. Following the manufacturer’s technique guide, the elbow was flexed 90° and adjusted to parallel the forearm and alignment bar attached to the target device. P1-P5 locking screws were inserted over the target device under direct visualization of the humerus.

**Figure 3:**
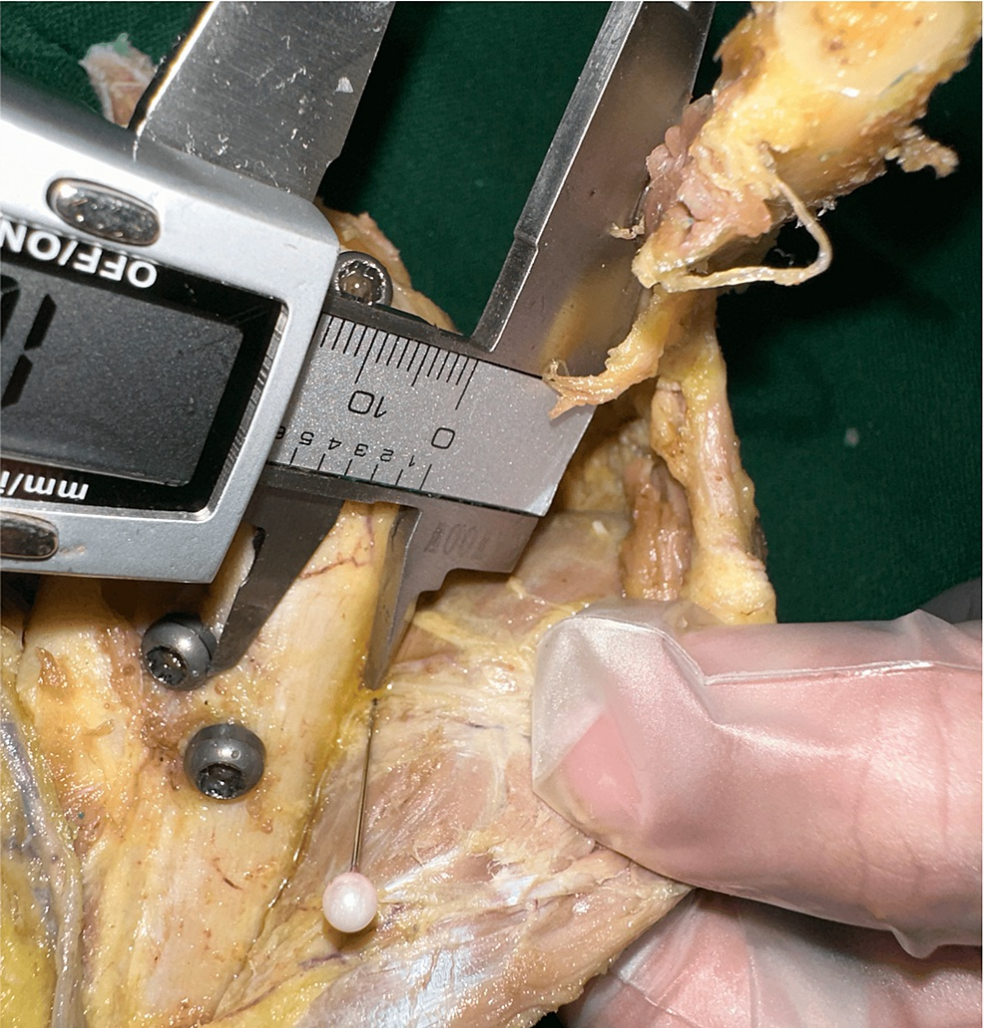
Measurement of the distance from the screw to the nerve The nerves were marked with a needle. The distance from the marked point to the outer edge of the screw head was measured with a digital caliper.

Digital calipers (GAWOOW Corporation, part number 001, JAN; 6950787196929) were used for all measurements. To achieve an accurate measurement of the distance between the medial support screw and each branch of the axillary nerve, we reduced the osteotomy sites of the clavicle and scapular spine once. The deltoid muscle was also reduced to its anatomical position with that reduction. The deltoid muscle was carefully turned over from anterior to posterior. The shortest distance between the medial support screw and each branch of the axillary nerve was measured with the arm in a neutral position of 0° rotation, abduction, and flexion (Figure 3). The distance of the top of the humeral head from the anterior axillary nerve was also measured with the arm in the neutral position. Two independent researchers performed the measurements twice to minimize inter- and intra-observer variation.

Statistical analysis

All statistical analyses were performed using Statistical Product and Service Solutions (SPSS, version 29.0; IBM Corp., Armonk, NY). A difference with P<0.05 was considered statistically significant. Descriptive statistics are presented as correlation coefficient, mean, percentage, minimum (min), and maximum (max). The correlation between the distance of each nerve from the medial support screws (P4 and P5) and humeral length was assessed using Spearman correlation analysis in a linear regression model. The level of correlation was interpreted as strong (correlation coefficient r=0.7-1), moderate (r=0.4-0.7), or low (r=0.2-0.4) depending on the degree of relationship after considering the value of significant correlation (P<0.05).

## Results

**Figure 4:**
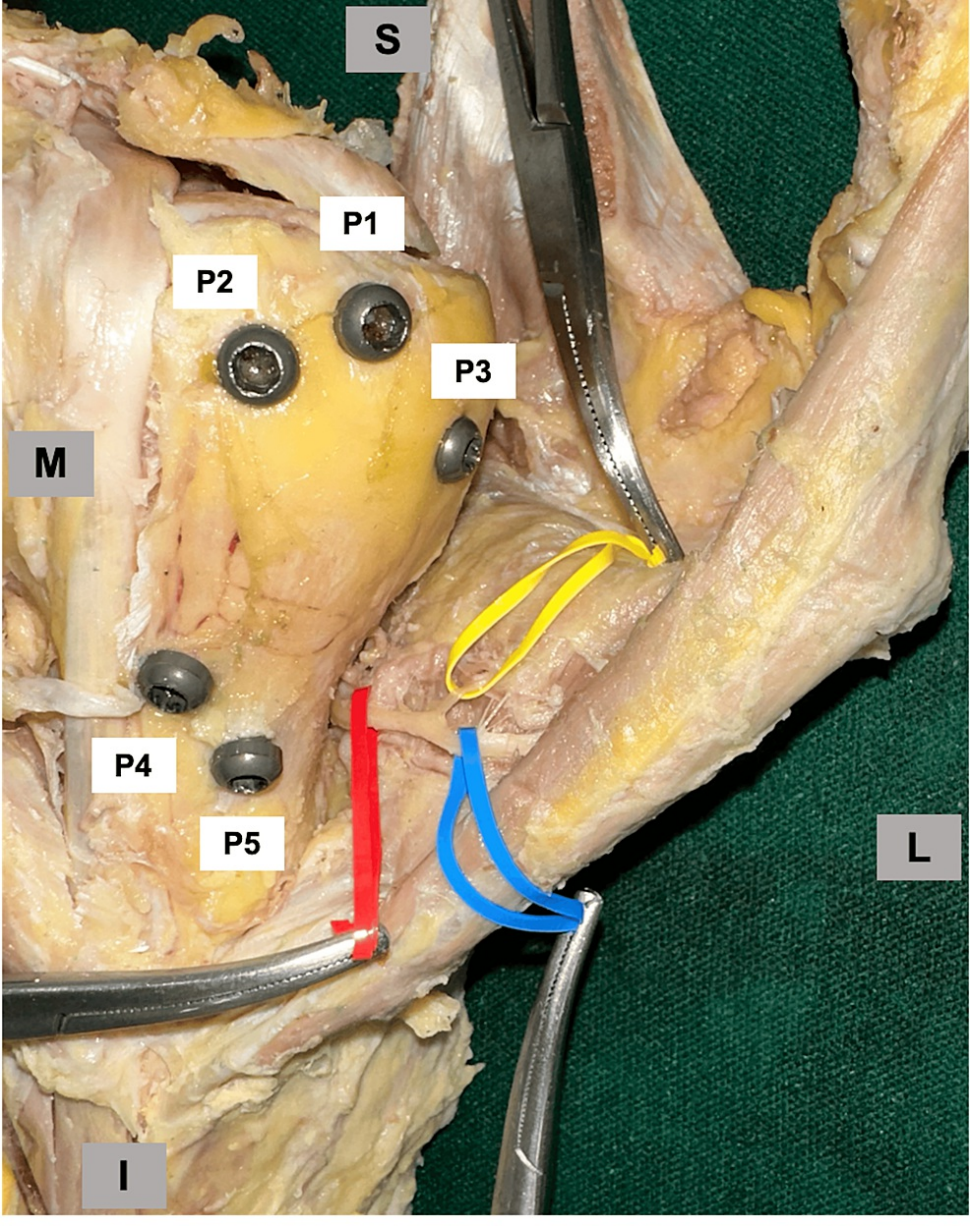
Location of the screws and nerves In the left cadaver shoulder, where the nerve is not in contact with the medial support screw, the anterior axillary nerve and its branches are located proximal to the medial support screws (P4, P5) and distal to the proximal locking screws (P1, P2, P3). Red tape: pre-AAN, yellow tape: ascending-AAN, blue tape: post-AAN

In 26 out of 29 shoulders, a bifurcation between the post-AAN and the ascending-AAN was observed. In the remaining three shoulders, this bifurcation could not be identified. In two (6.90%) of the 29 shoulders, the nerves came into contact with the medial support screws. In one case, the post-AAN was in contact with the P4 screw. In the other case, the post-AAN was in contact with the P4 screw, and the bifurcation between the post-AAN and the ascending-AAN was in contact with the P5 screw. In the remaining 27 cases (93.1%), the nerves were located proximal to the medial support screws (P4 and P5) (Figure 4).

The distance from the medial support screw to the anterior axillary nerve and its branches is shown in Table 1. The data in Table 1 were obtained from 26 of the 29 shoulders. The reason is that the post-AAN and ascending-AAN branches could not be identified in the remaining three shoulders. Table 2 shows the correlation between the distance of the anterior axillary nerve and its branches from medial support screws and the height and humeral length. Humeral length averaged 28.28 cm (range: 24.3-32.4 cm), and the distance from the humeral head apex to the anterior axillary nerve averaged 4.97 cm (range: 4.18-6.42 cm). There were no correlations between body height and the distance of the anterior branch of the axillary nerve and its branches from the medial support screws. The distance of P4 from pre-AAN showed a moderate correlation with humeral length (correlation coefficient: 0.421). However, the other parameters did not correlate significantly with humeral length. 

**Table 1:** Distance of the anterior axillary nerve and its branches from the medial support screw *Data were obtained from 26 of 29 shoulders. Data of the remaining three shoulders that did not show the post-AAN and ascending-AAN branches arising from the pre-AAN are not included. AAN: anterior axillary nerve

		Anterior branch of the axillary nerve and its branches		
		pre–AAN (mm)	post–AAN (mm)	ascending–AAN (mm)
Medial support screw	P4	20.45 ± 6.27 (range 5.62–34.2)	14.46 ± 5.61 (range 0–27.1)	16.93 ± 6.57 (range: 0–29.27)
	P5	15.65 ± 4.99 (range 6.5–28.1)	12.78 ± 5.09 (range 0–23.9)	15.61 ± 5.19 (range: 0–26.36)

**Table 2:** Correlation between the distance to each nerve and medial support screw and height and humerus length *P<0.056 is the significance level. AAN: anterior axillary nerve

		Height		Humeral length	
Medial support screw	Nerve	Correlation coefficient	P-value	Correlation coefficient	P-value
P4	Pre-AAN	0.32	0.11	0.421	*0.032
	Post-AAN	0.14	0.491	0.172	0.4
	Ascending-AAN	-0.03	0.885	-0.009	0.967
P5	Pre-AAN	0.33	0.098	0.357	0.073
	Post-AAN	0.043	0.836	-0.089	0.666
	Ascending-AAN	-0.011	0.956	-0.141	0.492

## Discussion

In this study, the anatomical relationship between the anterior branch of the axillary nerve and its branches and the medial support screw of the proximal humeral fracture nail was investigated in 29 shoulder cadavers. In two (6.90%) of the 29 shoulders, the branches of the anterior axillary nerve were in contact with the medial support screw. In approximately 90% of cases, those nerves were located proximal to the medial support screws. There was a moderate correlation between humeral length and the distance of pre-AAN from P4.

As the axillary nerve innervates the deltoid muscle, injury to this nerve significantly negatively affects shoulder function. Several studies have investigated potential axillary nerve palsy in classical proximal humeral fracture nails without medial support screws. Nijs et al. investigated the distance between the screw and the axillary nerve in six types of classical proximal humerus fracture nails. They showed a potential risk of iatrogenic axillary nerve palsy [14]. Prince et al. investigated the distance between the screw and the axillary nerve in four different classical proximal humeral fracture nails. They revealed that the oblique screw of the 9.5-mm Synthes short proximal humeral nail was at a potential risk of iatrogenic axillary nerve palsy [15]. In particular, the ascending-AAN showed the highest risk of injury. This study aligns with the findings from the previous study [15] since the pre-AAN was not in contact with the medial support screw. In addition, Spiegelberg et al. investigated the distance of the axillary nerve from the screw or blade of the antegrade intramedullary locking blade nail and showed that there was a high risk of iatrogenic axillary nerve palsy [16].

Medial support screws that improve mechanical stability and maintain fracture reduction are a revolutionary development in the treatment of proximal humeral fractures [1-5]. In recent years, new intramedullary nails with medial support screws have become available. However, no anatomical studies have investigated the risk of iatrogenic axillary nerve palsy in new intramedullary nails with medial support screws. Our study showed that, for the first time, there is a risk of injury to the anterior branch of the axillary nerve when intramedullary devices with medial support screws were used. Similar risks can occur with other proximal humeral fracture nails with medial support screws. Iatrogenic injury to the axillary nerve or its branches can cause deltoid muscle dysfunction.

To reduce the risk of axillary nerve injury, clinicians should perform a blunt dissection before inserting the instrument to make the path to the bone visible [15,16]. This study showed that, in approximately 90% of cases, the axillary nerve was located above the medial support screw. This suggests that, when inserting a medial support screw, the risk of axillary nerve injury may be reduced by inserting protection sleeves while retracting the anterior axillary nerve proximally with the deltoid muscle after a slightly inferior skin incision and blunt dissection.

Previous literature showed that the anterior branch of the axillary nerve is distributed an average of 5.8 cm (range: 4.8-7.5 cm) from the humeral head, with large individual differences [17]. The present study’s average was 4.97±0.57 cm (range: 4.18-6.42 cm). The discrepancy may be because all cadavers in this study were small East Asians.

Our study had several limitations. First, the sample size was small. Second, formaldehyde-fixed cadavers were used. Fresh cadavers would have been preferred for more accurate studies. Third, a single device was used. Comparisons with other devices may provide further insights. Fourth, no fluoroscopic devices were used. We understand the importance of controlling the height of the medial support screw under a fluoroscopic device. However, fluoroscopic devices were not available in the setting of the present anatomical study. Fifth, the ARISTO Nail is a device that is not available worldwide. However, we believe that this device, based on CT data from East Asians, is suitable for investigating the risk of potential axillary nerve injury in proximal humeral fractures in small Asian individuals.

## Conclusions

In this study, the potential risk of axillary nerve injury from medial support screws in intramedullary nails for proximal humeral fractures was investigated. Medial support screws in intramedullary nails for proximal humeral fractures had the potential to injure the axillary nerve and its branches. The anatomical findings of this study showed that upward traction of the soft tissue during insertion of the medial support screw may avoid iatrogenic damage to the anterior axillary nerve and its branches.
